# Work ability and associated factors in people living with human T-cell leukemia virus type 1

**DOI:** 10.1590/0037-8682-0111-2022

**Published:** 2022-08-05

**Authors:** Dayana Alves Costa, Fernando Martins Carvalho, Nicolle Melo Vieira, Gleicy Gabriela Spínola Carneiro Falcão, Viviane Almeida Sarmento, Carlos Brites, Liliane Lins-Kusterer

**Affiliations:** 1 Universidade Federal da Bahia, Programa de Pós-graduação em Medicina e Saúde, Salvador, BA, Brasil.; 2 Universidade Federal da Bahia, Faculdade de Medicina da Bahia, Salvador, BA, Brasil.; 3 Universidade Federal da Bahia, Faculdade de Odontologia, Salvador, BA, Brasil.

**Keywords:** human T-lymphotropic virus 1, Work capacity evaluation, Paraparesis, Tropical spastic, Quality of life

## Abstract

**Background:**

Infection with the human T-lymphotropic virus type 1 (HTLV-1) affects an estimated 10-15 million people worldwide. However, knowledge of the impact of HTLV-1 infection on work ability is lacking. This study aimed to measure the frequency and identify factors associated with poor work ability in patients living with HTLV-1.

**Methods:**

This cross-sectional study included 207 individuals infected with HTLV-1 who attended the University Hospital in Salvador, Bahia, Brazil. HTLV-1 antibodies were detected in the participants’ blood by enzyme-linked immunosorbent assay (ELISA) and confirmed by western blotting. Participants answered a questionnaire on sociodemographic data, personal habits, clinical data, health-related quality of life, and work ability, evaluated using the work ability index questionnaire. A Poisson regression model with a robust variance estimate was used to identify the factors associated with the prevalence of poor work ability.

**Results:**

Patients mean age was 55.2, ranging from 19 to 84 years, 73.0% were females, 100% had monthly family income less than US$ 394, and 33.8% presented HTLV-1 associated myelopathy/tropical spastic paraparesis (HAM/TSP). No individual was classified as having excellent work ability. Poor work ability prevalence was strongly associated (prevalence ratio; 95% confidence interval [CI]) with sedentarism (1.30; 1.03-1.65), neurological symptoms (1.25; 1.02-1.52), and low physical (0.95; 0.94-0.96) and mental (0.98; 0.97-0.99) component summaries of health-related quality of life.

**Conclusions:**

Poor work ability among people living with HTLV-1 is associated with sedentarism, neurologic symptoms, and low health-related quality of life.

## INTRODUCTION

Human T-lymphotropic virus type 1 (HTLV-1) is a type C retrovirus that was first isolated and identified from a patient with cutaneous T-cell malignancy in 1980^1^. It is transmitted through breastfeeding, sexual contact, blood transfusion, and sharing syringes and needles^2^. 

The prevalence of HTLV-1 infection is poorly known; however, it is estimated that it affects 10-15 million people worldwide^3^. Clusters of high prevalence were found in nearby areas with negligible prevalence. HTLV-1 infection is endemic in southwestern Japan, sub-Saharan Africa, South America, and the Caribbean area, with foci in the Middle East and Australo-Melanesia^4^. HTLV-1 infection is frequent in Brazil^5^, in the State of Bahia^6^, particularly in its capital, Salvador city, with an estimated prevalence of 1.8%^7^. 

Most people (approximately 95%) infected with HTLV-1 remain asymptomatic^8^. Individuals with HTLV-1 have a 57% greater risk of death due to any cause than those HTLV-1-negative individuals. HTLV-1 is associated with increased odds of seborrheic dermatitis and Sjogren’s syndrome and a lower relative risk of gastric cancer^9^. HTLV-1 can cause two severe diseases, adult T-cell leukemia/lymphoma (ATLL) and HTLV-1-associated myelopathy/tropical spastic paraparesis (HAM/TSP). It is estimated that 0.25-3% of people infected with HTLV-1 will develop HAM/TSP during their lifetime. HAM/TSP mainly occurs in adulthood. HAM/TSP has an insidious onset that progressively evolves to neurological features, such as spasticity or hyperreflexia of the lower extremities, lower extremity muscle weakness, and urinary bladder disturbances. Approximately 50% of cases present with sensory disturbances and low back pain. HAM/TSP can be associated with other HTLV-1 associated symptoms like uveitis, myositis, and infective dermatitis^10^. Patients with HAM/TSP have difficulty performing daily routine activities, particularly because of a disturbed gait that compromises physical, emotional, and social aspects, impairing their quality of life^11^. Patients with HIV-HTLV-1 coinfection or HTLV-1 infection report more difficulty performing daily activities than those with exclusive infection with human immunodeficiency virus (HIV)^12^. 

The work ability index (WAI) questionnaire is an instrument that evaluates workers’ perception of work demands and the environment, work organization, work community, promotion of workers’ health and functional capacity, and promotion of professional competence. Good work ability means high-quality work, enjoyment of staying in one’s job, and the expectation of a meaningful retirement^13^. 

Therefore, this study aimed to measure the frequency and identify factors associated with work ability in patients living with HTLV-1. 

## METHODS

### Study design and study population

This cross-sectional study was conducted from February 2018 to December 2019 at the University Hospital, Federal University of Bahia, Brazil. This study is part of broader research that investigates other health aspects of people with HTLV-1^14^. The target population comprised 209 individuals aged 18 years or older who were invited to participate in the study. Severe cognitive deficits that prevented the elicitation of reliable information in the interview were an exclusion criterion. There were only two refusals, resulting in a final study population of 207 individuals.

### Data collection instruments and procedures

Participants were interviewed by a member of the research team after the medical consultation in a quiet room, keeping the patient’s privacy. Information about sociodemographic characteristics (age, race, schooling, civil status, number of children, and monthly family income - coded as < 1 Brazilian minimum wage and 1-2 Brazilian minimal wage. One Brazilian minimal wage was equivalent to US$ 197 by the time of the study) personal habits (smoking, drinking, and sedentarism), health-related quality of life, clinical data (comorbidities and HTLV-1 symptoms), and work ability were collected using structured questionnaires. 

Work ability was used as the dependent variable. Work ability was evaluated using the WAI questionnaire. WAI is a summary measure of seven dimensions (range 7-49): 1 -current work ability compared with the lifetime best, 2 - work ability in relation to the demands of the job, 3 - number of current diseases diagnosed by a physician, 4 - estimated work impairment due to diseases, 5 - sick leave, 6 - self-prognosis of work ability 2 years from now, and 7 - mental resources. The total score was classified into four work ability categories: poor (7-27 points), moderate (28-36 points), good (37-43 points), and excellent (44-49 points). For the purposes of this study, the four possible subgroups of WAI were categorized as poor versus others^15^. The WAI questionnaire was validated in a Brazilian population and showed satisfactory psychometric properties^16^.

**FIGURE 1:**
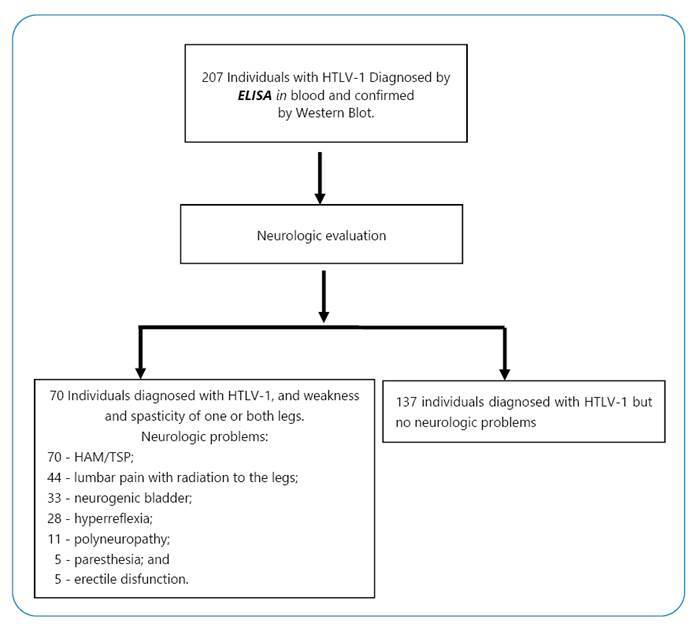
Study population flowchart.

Neurologic evaluation was performed on all 207 individuals at the University Hospital, according to the World Health Organization criteria^17^. Seventy (33.8%) of the 207 individuals in the study presented neurological symptoms; all of them presented weakness and spasticity of one or both legs, compatible with HAM/TSP. Other diagnostics were 44 lumbar pain, 33 neurogenic bladder, 28 hyperreflexia, 11 polyneuropathy, and 5 erectile dysfunction (Figure 1).

Health-related quality of life was evaluated using the 36-Item Short-Form Health Survey version 2 (SF-36v2) questionnaire. This instrument comprises 36 items that generate eight domains: physical functioning, physical role, bodily pain, general health, vitality, social functioning, emotional role, and mental health. Two summary measures can be calculated from these domains: physical and mental component summaries. The psychometric properties of the SF-36v2 have been validated in a Brazilian population^18^. PRO CoRE software, version 1.4 (Optum Inc., Johnston, RI, USA), was used to score the survey. The normalized scores have a mean of 50 and a standard deviation of 10, a transformation that enables better comparisons among domains. A commercial license (license number QM025905) granted permission for using the SF-36v2. 

### Laboratory examinations

HTLV-1 antibodies were detected in the blood of the participants by enzyme-linked immunosorbent assay (ELISA) and confirmed by western blotting at the Infectology Research Laboratory, University Hospital, Federal University of Bahia.

### Statistical data analysis

Differences between subgroups of continuous variables were compared using Student's t-test. Differences between subgroups of categorical variables were compared using Pearson's chi-square test. Variables with a *P*-value < 0.20 in bivariate analysis were selected for composing a Poisson regression model with robust variance estimators that had work ability as the dependent variable^19^ ^-^ ^21^. Cronbach’s alpha coefficient was used to evaluate the internal consistency of the SF-36v2 and WAI instruments; values above 0.70 were considered acceptable^22^ ^-^ ^23^. 

### Ethical aspects

The research protocol was approved by the research ethics committee of the Federal University of Bahia (opinion number:30762714.4.0000.5577). All the participants provided written informed consent. 

## RESULTS

The mean age was 55.2, ranging from 19 to 84 years, 73.0% were females, 100% had a monthly family income less than US$ 394, and 33.8% presented HAM/TSP. The work ability of 207 individuals with HTLV-1 was poor in 54.1% (n = 112), moderate in 37.7% (n = 78), and good in 8.2% (n = 17). No individual was classified as having excellent work ability. The alpha coefficient of the WAI questionnaire was 0.84, indicating a high internal consistency.

The poor work ability prevalence rate was significantly higher (*P* < 0.20) among individuals who had children, had schooling < 8 years, civil status other than stable relation, who did not referred alcohol consumption, sedentary status, comorbidities, and neurological symptoms (Table 1).

**TABLE 1:** Work ability according to characteristics of 207 individuals with HTLV-1, Salvador, Brazil, 2018-2019.

	Work ability							
	Poor		Moderate/good				
	(n = 112)		(n = 95)				
Characteristic	n	%	n	%	PR	95% CI	P-value
Sex							
Male	31	55.4	25	44.6	1.03	0.78-1.36	0.826
Female	81	53.4	70	46.6			
Race							
White	10	58.8	7	41.2	1.10	0.72-1.67	0.683
Other	102	53.7	88	46.3			
Children							
No	11	39.3	17	60.7	0.70	0.43-1.12	0.090
Yes	101	56.4	78	43.6			
Schooling							
< 8 years	80	61.1	51	38.9	1.45	1.08-1.95	0.008
≥ 8 years	32	42.1	44	57.9			
Civil status							
Other	66	58.9	46	41.1	1.22	0.94-1.58	0.131
Stable relation	46	48.4	49	51.6			
Monthly family income							
< 1 MW	36	55.4	29	44.6	1.04	0.79-1.35	0.803
1 to 2 MW	76	53.5	66	46.5			
Smoking							
Yes	11	68.8	5	31.2	1.30	0.91-1.86	0.222
No	101	52.9	90	47.1			
Drinking							
Yes	22	42.3	30	57.7	0.73	0.52-1.03	0.049
No	90	58.1	65	41.9			
Comorbidities							
Yes	97	59.1	67	40.9	1.70	1.11-2.60	0.005
No	15	34.9	28	65.1			
Sedentary							
Yes	79	57.7	58	42.3	1.22	0.92-1.63	0.151
No	33	47.1	37	52.9			
Neurological symptoms							
Yes	55	78.6	15	21.4	1.89	1.50-2.38	< 0.001
No	57	41.6	80	58.4			

*Fisher test; **MW:** Brazilian minimal wage (approx. 197.39 US$/month).

Bivariate analyses showed that individuals with poor work ability presented systematically lower (*P* < 0.001) SF-36 domain scores and physical and mental component summaries of health-related quality of life and were significantly older (*P* < 0.042) than those with moderate or good work ability (Table 2).

**TABLE 2:** Work ability according to SF-36 health-related quality of life domains (mean [SD], in %) and age (mean [SD], in years) of 207 individuals with HTLV-1, Salvador, Brazil, 2018-2019.

		Work ability		
		Poor	Moderate/good	
Variable	Cronbach alpha	(n = 112)	(n = 95)	***P*-value**
Physical Functioning	0.95	33.1 (10.9)	50.9 (8.9)	< 0.001
Role Physical	0.95	29.1 (10.4)	48.4 (12.9)	< 0.001
Bodily Pain	0.78	34.6 (11.2)	47.5 (11.1)	< 0.001
General Health	0.77	36.2 (10.0)	50.2 (8.9)	< 0.001
Vitality	0.82	41.6 (12.7)	56.2 (10.0)	< 0.001
Social Functioning	0.75	38.3 (14.3)	52.9 (8.9)	< 0.001
Role Emotional	0.94	32.8 (16.6)	49.0 (13.6)	< 0.001
Mental Health	0.84	39.1 (15.5)	51.6 (10.3)	< 0.001
Physical Component Summary	-	32.7 (9.7)	49.1 (9.8)	< 0.001
Mental Component Summary	-	40.3 (16.6)	52.5 (10.7)	< 0.001
Age	-	56.8 (12.4)	53.3 (12.4)	0.042

The Poisson regression model estimated that adjusted prevalence rates (PR) of poor work ability were 30% higher among sedentary individuals (PR = 1.30; 95% confidence interval [CI]: 1.03-1.65) and 25% higher among those with neurological symptoms (PR = 1.25; 95% CI: 1.02-1.52). The mean level of the physical component summary of the health-related quality of life was 5% lower (PR = 0.95; 95% CI: 0.94-0.96), and the mean level of the mental component summary was 2% lower (PR = 0.98; 95% CI: 0.97-0.99) among individuals with poor work ability compared with those with moderate or good work ability. The alpha coefficients of the eight domains of the SF36v2 questionnaire varied from 0.75 to 0.95, revealing high internal consistency (Table 3).

**TABLE 3:** Results of Poisson regression having the prevalence ratio of low work ability as the dependent variable among 207 individuals with HTLV-1, Salvador, Brazil, 2018-2019.

Predictors (referent)	PR	95% CI	***P*-value**
Children (Yes)	0.91	0.66-1.26	0.571
Schooling (≥ 8 years)	1.05	0.84--1.31	0.696
Civil status (Stable relation)	1.07	0.87-1.31	0.502
Drinking (No)	1.12	0.86-1.46	0.392
Comorbidities (No)	0.98	0.69-1.35	0.845
Age (Years)	1.01	1.00-1.02	0.061
Sedentary (No)	1.30	1.03-1.65	0.030
Neurological symptoms (No)	1.25	1.02-1.52	0.028
Physical Component Summary (%)	0.95	0.94--0.96	< 0.001
Mental Component Summary (%)	0.98	0.97-0.99	< 0.001

**PR:** adjusted prevalence ratio.

## DISCUSSION

Poor work ability was common in the study population (54.1%). In addition, poor work ability is associated with an increased risk of sickness absence^24^, early retirement^25^, and higher mortality in older age^26^.

This study among people living with HTLV-1 found that poor work ability was associated with sedentarism, neurologic symptoms, and low health-related quality of life in both the physical and mental components.

Multivariate analysis estimated that the adjusted prevalence of poor work ability was 30% higher among individuals with a sedentary lifestyle and 25% higher among those presenting with neurologic symptoms. People infected with HTLV-1, who already present with neurological symptoms, are expected to have impaired work ability. Patients with HAM/TSP usually have impaired gait, dependence on daily activities, and a poor quality of life due to intense muscle weakness^27^. The same reasoning applies to the relationship between sedentarism and poor work ability^28^. Unfortunately, this study did not collect information on the temporal sequence of the relationship between these independent variables (sedentarism and neurologic symptoms) and outcomes (work ability).

HTLV-1 infection has been associated with several diseases. Fortunately, only a few of these are fatal, such as leukemia. However, this disease is rare and has a relatively low impact on the community mortality rates^9^. The results of this study raise awareness of the poorly recognized burden of HTLV-1 infection on the morbidity caused by neurologic symptoms and its impact on work ability.

Individuals with poor work ability had a lower health-related quality of life than those with moderate or good work ability. The differences found in the bivariate analyses were confirmed in the multivariate analyses, which estimated a 5% lower physical and a 2% lower mental component summary for patients with poor work ability after adjusting for relevant variables. The complex construct of the WAI questionnaire has many points of convergence with that of the SF36v2^29^ since both deal with physical and mental demands. Therefore, the WAI score is expected to be strongly associated with SF36v2 dimensions and component summaries^30^ ^,^ ^31^. For example, the progressive and disabling gait disturbances of patients with HTLV-1 may impair physical, emotional, social, and mental aspects that, in turn, may modify the health-related quality of life perception^11^.

The magnitude of the differences in physical and mental component summaries according to work ability can be analyzed from the perspective of the minimal clinically important difference (MCID)^32^. The concept of MCID evolved to minimal important difference, defined as “the smallest difference in score in the domain of interest that patients perceive as important, either beneficial or harmful, and that would lead the clinician to consider a change in the patient’s management^33^.”

The MCID for physical component summary varied in studies, including patients with moderate to severe psoriasis (2.5 points)^34^, undergoing lumbar spine surgeries (4.11-5.21^35^; and 4.93)^36^, and surgical (7.83) and non-surgical (2.15) patients with spinal deformities^37^. The MDIC for mental component summary was 2.5 points in a study of patients with moderate to severe psoriasis^34^. Roughly half of all patients treated for hepatitis C fail to achieve clinically important improvements in physical and mental component summaries^38^. However, the MCID is not an immutable characteristic and may vary by population and context^39^. Concerning patients’ quality of life scores, the MCID for group-level is necessarily smaller than those for individual patient-level^40^. We are unaware of a study that determined the MCID for the work ability index among patients with non-alcoholic fatty liver disease (NAFLD), similar to our study population. 

The high frequency (54.1%) of poor work ability among patients with NAFLD and the nature of the factors associated with poor work ability suggest the need to implement strategies to provide adequate health care among people living with HTLV-1.

One important limitation of this preliminary cross-sectional study is the lack of information about the temporal sequence of neurological symptoms and sedentarism related to the investigated outcomes and poor work ability. However, to the best of our knowledge, this is the first study to evaluate work ability and associated factors among people living with HTLV-1. 

The frequency of poor work ability among people living with HTLV-1 was high and was associated with sedentarism, neurologic symptoms, and low health-related quality of life.
